# The Phosphoinositide 3-Kinase p110α Isoform Regulates Leukemia Inhibitory Factor Receptor Expression via c-Myc and miR-125b to Promote Cell Proliferation in Medulloblastoma

**DOI:** 10.1371/journal.pone.0123958

**Published:** 2015-04-27

**Authors:** Fabiana Salm, Valeriya Dimitrova, André O. von Bueren, Paulina Ćwiek, Hubert Rehrauer, Valentin Djonov, Pascale Anderle, Alexandre Arcaro

**Affiliations:** 1 Department of Clinical Research, Division of Pediatric Hematology/Oncology, University of Bern, Bern, Switzerland; 2 Department of Pediatrics and Department of Pediatric Hematology and Oncology, Georg August University Goettingen, Goettingen, Germany; 3 Functional Genomics Center Zurich, ETH and University of Zurich, Zurich, Switzerland; 4 Institute of Anatomy, University of Bern, Bern, Switzerland; 5 Institute of Biochemistry and Molecular Medicine, University of Bern, Swiss National Centre of Competence in Research TransCure, University of Bern, Bern, Switzerland; 6 Swiss National Centre of Competence in Research Molecular Oncology, Swiss Institute for Experimental Cancer Research, Ecole Polytechnique Fédérale de Lausanne, School of Life Sciences, Lausanne, Switzerland; 7 Swiss Institute of Bioinformatics, Lausanne, Switzerland; Shriners Hospitals for Children, UNITED STATES

## Abstract

Medulloblastoma (MB) is the most common malignant brain tumor in childhood and represents the main cause of cancer-related death in this age group. The phosphoinositide 3-kinase (PI3K) pathway has been shown to play an important role in the regulation of medulloblastoma cell survival and proliferation, but the molecular mechanisms and downstream effectors underlying PI3K signaling still remain elusive. The impact of RNA interference (RNAi)-mediated silencing of PI3K isoforms p110α and p110δ on global gene expression was investigated by DNA microarray analysis in medulloblastoma cell lines. A subset of genes with selectively altered expression upon p110α silencing in comparison to silencing of the closely related p110δ isoform was revealed. Among these genes, the leukemia inhibitory factor receptor α (LIFR α) was validated as a novel p110α target in medulloblastoma. A network involving c-Myc and miR-125b was shown to be involved in the control of LIFRα expression downstream of p110α. Targeting the LIFRα by RNAi, or by using neutralizing reagents impaired medulloblastoma cell proliferation *in vitro* and induced a tumor volume reduction *in vivo*. An analysis of primary tumors revealed that LIFRα and p110α expression were elevated in the sonic hedgehog (SHH) subgroup of medulloblastoma, indicating its clinical relevance. Together, these data reveal a novel molecular signaling network, in which PI3K isoform p110α controls the expression of LIFRα via c-Myc and miR-125b to promote MB cell proliferation.

## Introduction

Medulloblastoma is the most common malignant brain tumor in children and accounts for approximately 20% to 25% of all pediatric central nervous system tumors [1, 2]. Most medulloblastoma occur between 5 and 10 years of age [1, 3]. The standard treatment of medulloblastoma involves surgery followed by chemotherapy and, in the case of children older than 3–5 years, craniospinal radiotherapy [3]. However, nearly half of all patients die from progressive disease and the 5-year survival rate is below 50% for high-risk patients [2]. There is an urgent need to develop novel targeted therapeutic approaches for medulloblastoma, which will originate from a better understanding of the disease biology.

Medulloblastoma has been recognized to be a heterogeneous disease, and no recurrent cancer gene mutations have been found, although many of the mutations described so far affect key intracellular signaling pathways, such as sonic hedgehog (SHH) and Wnt/β-catenin [4, 5]. Genomic studies in large series of primary medulloblastoma have led to a novel classification of medulloblastoma into different molecular subtypes, characterized by the activation of sonic hedgehog (SHH), Wnt/ β-catenin or c-Myc pathways (Group 3) [5]. c-Myc plays an important role in medulloblastoma biology and therefore targeting the signaling networks controlled by c-Myc may be a promising approach to develop targeted therapies for the subsets of tumors in which c-Myc is activated [6–11].

Another signaling pathway which is being considered to develop targeted therapies for medulloblastoma, is the receptor tyrosine kinase (RTK) cascade, which links polypeptide growth factors to cellular responses via their downstream signaling intermediates, in particular phosphoinositide 3-kinase (PI3K), Akt, the mammalian target of rapamycin (mTOR) and mitogen-activated extracellular signal-regulated kinase activating kinase (MEK) [12–18].

The PI3K signaling pathway controls key cellular responses, such as cell growth and proliferation, survival, migration and metabolism. Over the last decades, it has been recognized that this intracellular signaling pathway is frequently activated by genetic and epigenetic alterations in human cancer, including malignant brain tumors [4, 15, 19, 20]. The PI3K family of signaling enzymes comprises eight catalytic isoforms, which are subdivided into three classes. The class I_A_ PI3K isoform p110α is considered to be a validated drug target in human cancer, in particular because activating mutations in the *PIK3CA* frequently occur in human cancer [21, 22]. In medulloblastoma, the *PIK3CA* gene is targeted by mutations at a low frequency [23], but the p110α isoform is also over-expressed in primary tumors and cell lines [15]. Our previous studies demonstrate the distinct roles of the class IA PI3K isoforms in medulloblastoma, from which p110α showed the strongest effects in the control of medulloblastoma proliferation, survival and chemoresistance. Targeting p110α by RNAi or isoform-specific inhibitors impaired medulloblastoma cell proliferation, survival and chemoresistance, while similar effects were not observed for p110δ. However, co-targeting of p110α and p110δ led to increased effects on medulloblastoma cell proliferation [15].

Clinical trials have started to evaluate the safety and efficacy of agents targeting this pathway in different brain tumors including medulloblastoma [19]. However, targeting the PI3K pathway remains challenging. Thus, the effort of characterizing the molecular mechanisms and the PI3K downstream effectors in MB will contribute to the elucidation of how PI3K drives oncogenic signaling and may lead to the identification of PI3K target genes as novel candidates for targeted therapy in MB.

Here, we performed a genomic study to compare the changes in the global gene expression profiles of medulloblastoma cells caused by RNAi-mediated down-regulation of p110α or p110δ. Among both *PIK3CA* and *PIK3CD* responsive genes, we identified c-Myc as the transcription factor, whose network of genes was mostly deregulated. Intriguingly, the c-Myc network included the α-subunit of the receptor for the leukemia inhibitory factor (LIFRα). Our data describe, for the first time, a signaling network in which c-Myc controls the expression of LIFRα, in part through the regulation of miR-125b, to contribute to oncogenic p110α signaling in medulloblastoma.

## Materials and Methods

### Cell culture and treatments

The MB cell lines were obtained and cultured as described in [15], the stable clones DAOY V11 (empty vector transfected) and DAOY M2.1 (*MYC* vector transfected) were described in [24, 25]. PFSK and PNET5 medulloblastoma cell lines were purchased from the American Type Culture Collection and were grown in RPMI 1640 with 10% FCS and penicillin/streptomycin/L-glutamine (Sigma, Buchs, Switzerland). D341 and D458 were a kind gift of Dr. Henry Friedman (Duke University, Durham, NC) [26] and were cultured in Improved MEM medium (Invitrogen, Carlsbad, CA, USA) supplemented with 1% L-glutamine, 1% Penicillin/Streptomycin, 10% FCS. The UW228 cells expressing tamoxifen-inducible c-Myc-ER were kindly provided by Prof. Annie Huang, Hospital for Sick Children, Toronto, Canada and described in [27]. All cells were grown in a humidified atmosphere at 37° and 5% CO2. Tamoxifen (Sigma) was used to induce c-Myc expression in these clones. The PI3K inhibitors PIK75 and YM024 (Calbiochem, Darmstadt, Germany) were dissolved in DMSO (Sigma) at 10 mM and diluted to the indicated concentrations in cell culture medium just before use. The Anti-human LIFRα antibody (AF-249-NA) and the recombinant rh-LIF Rα (7487-LR) were purchased from R&D Systems (Minneapolis, MN, USA) and were diluted directly into the medium immediately before use. For growth factor stimulations, cells were grown to confluence, starved overnight in culture medium containing 1% FCS. Cells were maintained in serum-free RPMI for 1 h and were then stimulated with LIF (Sigma, Buchs, Switzerland) for 10 min.

### RNA interference and miRNA transfection

MB cell lines were transfected with siRNA pools, each comprising four individual oligonucleotides (SMARTpool small interfering RNA reagents; Dharmacon, Waltham, MA, USA), directed against *PIK3CA* (M-003018-0), *PIK3CD* (M-006775-02-0005), *MYC* (M-003282-07), *LIFR* (M-008017-01-0005) using Lipofectamine 2000 (Invitrogen, Carlsbad, CA, USA) as directed by the manufacturer for adherent cell lines. siCONTROL Non-targeting siRNA Pool (D-001206-14-20) (Dharmacon) composed of four siCONTROL Non-targeting siRNAs was used as negative control. All siRNAs were used at a final concentration of 20 nM. Cells were incubated for 24 h to 48 h and after mRNA and protein were extracted to assess protein expression by Western blotting and mRNA expression by TaqMan analysis.

DAOY cells were transfected with 20nM of miRIDAN hairpin inhibitor directed against mature hsa-miR-125b, 20 nM of precursor for hsa-miR-125b-5p or with Hairpin Negative Control (Thermo Fisher Scientific, Waltham, MA, USA) using SilentFect (Biorad, Hercules, CA, USA) as suggested by the manufacturer. Protein and mRNA were extracted and analyzed after 48h.

### Plasmid transfections

DAOY cells were transfected with expression plasmids using the Lipofectamine Plus reagent (Invitrogen) according to the manufacturer's recommended protocol. The plasmids encoding for activated PI3K-p110α (PIK3CA CAAX) and pcDNA3 (empty vector control) were described in [28].

### Microarray analysis

The cDNA microarray analysis was performed in collaboration with the Functional Genomic Center of the University Zurich, Switzerland. Human Genome-U133 Plus 2.0 Affymetrix GeneChips arrays (Affymetrix, Santa Clara, CA, USA) were used to assess the gene expression data. Raw data generated by the GCOS Software (Affymetrix) were processed by using the RMA method [29] and further statistically analyzed by using the software R and applying Student’s t-test.

Each experiment represented a group of three independent biological replicates. Results are expressed as fold change, and differences in expression were considered significant if the fold change was > 2.0 or <-2 and the P-value < 0.001. Comparative and cluster analysis of the data were performed using the softwares Cluster 3.0 and TreeView [30]. The GeneGO MetaCore (GeneGO, St Joseph, MI, USA) was used to identify affected pathways and networks of genes, according to their ontological categories. The data was deposited in the Gene Expression Omnibus and is accessible through GEO series accession number GSE40564 (http://www.ncbi.nlm.nih.gov/geo).

### Gene expression analysis

Total RNA was extracted using the RNeasy Mini Kit (Qiagen, Basel, Switzerland) and converted into cDNA using High-Capacity cDNA Reverse Transcription Kit according to manufacturer's instructions (Applied Biosystems, Foster City, CA, USA). Assays-on-Demand Gene Expression products (Applied Biosystems) were used to measure mRNA expression levels of *PIK3CA* (Hs00907965_m1), *PIK3CD* (Hs00192399_m1), *MYC* (Hs00153408_m1), *LIFR* (Hs00158730_m1) and *GAPDH* (Hs99999905_m1; internal control gene). Relative mRNA expression levels were calculated using the comparative threshold cycle (CT) method.

The miRNAs extraction procedure via RNeasy Mini Kit (Qiagen, Basel, Switzerland) was followed by specific reverse transcription and amplification using hsa-miR-125b (000449) and U6 snRna (001973; loading control). Relative miRNA expression levels were calculated using the comparative threshold cycle (CT) method.

For semi-quantitative analysis of the gene expression, the One-Step RT-PCR Kit (Qiagen) was employed. The primers for *LIF*, *LIFR* and *GAPDH* were purchased from Microsynth (Balgach, Switzerland). DNA was separated on 2.5% agarose gel and stained with GelRed (Biotium, Hayward, CA, USA).

The expression data of medulloblastoma (n = 62) which are available from NCBI’s Gene Expression Omnibus (http://www.ncbi.nlm.nih.gov/geo/; accession number GSE10327) published by Kool et al., [31] and expression data of a second cohort of medulloblastoma (n = 103; available from NCBI’s Gene Expression Omnibus (http://www.ncbi.nlm.nih.gov/geo/; accession number GSE21140, reported by Northcott et al. [32]) were used for analysis. Data are accessible through the open access database R2 for visualization and analysis of microarray data (http://r2.amc.nl), and data were generated using the R2 microarray analysis and visualization platform (http://r2.amc.nl). Gene expression levels were compared using the Kruskal-Wallis test. If this global test was significant, Dunn's post hoc tests were used to compare the groups.

### Western blotting

Cell lysates were prepared in RIPA buffer (50mM Tris-Cl, [pH 6.8], 100mM NaCl, 1% w/v Triton X-100, 0.1% w/v SDS) supplemented with Complete Mini Protease Inhibitor Cocktail (Roche Applied Sciences, Germany) and with the phosphatase inhibitors β-glycerophosphate (20mM) and Na_3_VO_4_ (200 mM). Proteins were separated by SDS–polyacrylamide gel electrophoresis and transferred on hydrophobic polyvinylidene difluoride (PVDF) membrane (Amersham, GE Healthcare, UK). Antibodies specific for PI3K p110α (sc-1331), p110δ (sc-136032), c-Myc (sc-789), LIFR (sc-659), Akt1/2/3 (sc-8312), ERK 1/2 (sc-99) (Santa Cruz Biotechnology, CA, USA) were diluted 1/500 in 5% BSA, phospho-ERK1/2 (Thr202/Tyr204) (cs-4370), phospho-Akt (Ser473; Thr308) (cs-3787; cs-2965), phospho-S6 (Ser235/236, Ser240/244) (cs-2211; sc-5364) (Cell Signaling Technology, Inc., Danvers, MA, USA), β-actin (Sigma-Aldrich Chemie GmbH, Buchs, Switzerland) (A5441) were diluted 1/1000 in 5% BSA. Chemiluminescence was used for detection using SuperSignal West Femto Maximum Sensitivity Substrate (Thermo Fisher Scientific Inc., Rockford, IL, USA).

### Cell proliferation assays

Cell proliferation was assessed using the Cell Titer 96 Aqueous Cell Proliferation Assay (Promega, Madison, WI, USA). Data are expressed as average values from at least three independent experiments.

### **Chicken chorioallantoic membrane assa**y

Fertilized chicken eggs (gallus gallus) purchased from a local hatchery were incubated in a humidified incubator at 37°C. On embryonic day 3, a squared opening was excised into the shell and sealed with tape. Upon 96h of incubation, a silicon ring was placed on the chorioallantoic membrane and after gentle a scraping, a suspension of 5 million DAOY cells in 20ml phosphate-buffered saline was applied onto the lacerated membrane. On embryonal day 10, the formed tumors were treated with either rh-LIFα (Recombinant Human LIFRα, R&D Systems, Minneapolis, USA, catalog number 7487-LR) or phosphate-buffered saline for 4 consecutive days. On embryonal day 14, tumor volume was measured, as described in [33] and the embryos were sacrificed.

### ChIP-on-chip

ChIP-on-chip experiments using a MYC antibody were performed as previously described [24, 34].

### Ethics statement

All the experiments were performed in conformity with the Swiss animal protection law, requiring no specific approval for working on avian embryos before day 15 of their embryonal development. Thus, on day 14, the experiments were terminated by arterial excision.

### Statistical analysis

All experiments were performed at least in triplicates. Data are represented as mean ± SD. The statistical significance of differences between groups was assessed with ANOVA using Tukey’s post hoc tests calculated with the statistical software GraphPad PRISM 5 (GraphPad Software, San Diego, CA, USA); P-Values of <0.05 were considered significant and indicated with a single asterisk, a double asterisk if P <0.001 or a triple asterisk if P <0.001. All statistical analyses were intended to be rather exploratory than confirmatory and nominal p-values are reported, without adjustment for multiple testing.

Distribution of gene expression levels were compared using the Kruskal-Wallis test. P-values < 0.05, two-tailed were considered statistically significant. Statistical analyses were performed using GraphPad Prism 4 (GraphPad Software, San Diego, CA, USA).

## Results

### Gene expression analysis reveals selective gene subsets regulated by p110α

**Fig 1:**
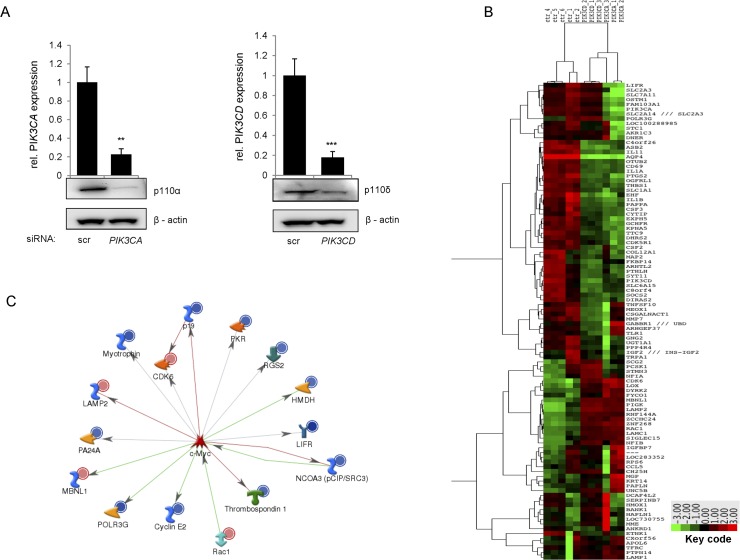
Gene expression analysis. (a) DAOY cells were transfected with siRNA against *PIK3CA* or *PIK3CD* and analyzed 48h post transfection for gene down-regulation at mRNA level by real-time PCR and at protein level by Western blot. (b) Heat map representing the expression of *PIK3CA* and *PIK3CD* regulated genes. (c) GeneGo Metacore analysis of transcriptional networks. The set of genes deregulated by *PIK3CA* silencing in DAOY cells were analyzed via GeneGo Metacore software. The most affected network comprising the oncogene c-Myc and some its downstream targets is shown.

In order to investigate how the class I_A_ PI3K isoforms p110α and p110δ regulate the expression of specific gene subsets in medulloblastoma, we performed cDNA microarray analysis in DAOY cells transiently transfected with either siRNA targeting p110α, p110δ or control siRNA (Fig 1B). The efficacy of the down-regulation of the target proteins by the respective siRNAs was demonstrated by Western blot analysis, as well as quantitative real time PCR 48h post transfection (Fig 1A). Thus at 48h post transfection, the most efficient target down-regulation was observed and it was therefore chosen for the cDNA microarray analysis. Each experiment was performed in three independent biological replicates, where the results were normalized to the control values and expressed as fold changes. To define lists of differentially expressed genes, we considered significant those whose fold change was > 2.0 or <-2 and the P-value < 0.001. Silencing p110α significantly affected the expression of 453 genes, while silencing p110δ resulted in significant changes in 305 genes, showing that p110α has an overall higher impact on the regulation of global gene expression in MB. In addition, 75 genes were commonly regulated by both p110α and p110δ (S1 and S2 Tables). The resulting sets of differentially expressed genes were further analyzed using different bioinformatics tools, in order to establish functional networks and identify relevant targets for validation.

### Silencing of p110α perturbs multiple transcriptional networks in medulloblastoma

To gain insight into the transcriptional networks affected by silencing of p110α and p110δ in medulloblastoma cells, we performed a biostatistics analysis of the gene expression data with GeneGO MetaCore. The software was used to define functional annotations for the selected players, thus assigning them to ontologic categories for association with relevant biologic processes and pathways. The transcriptional networks that were most significantly altered by the down-regulation of p110α and p110δ comprised c-Myc, the estrogen receptor (ER), p53 and STAT3 (S3 Table). The transcriptional network involving c-Myc included the LIFRα as a target gene (Fig 1C). LIFRα was found to be more significantly down-regulated in DAOY cells transfected with p110α siRNA, than in the case of p110δ siRNA (Fig 1B and S4 Table), suggesting that it is selectively regulated by p110α.

Interestingly, STAT3, a downstream target of LIFR was identified as one of the most significantly altered networks (S3 Table). Thus, down-regulation of p110α perturbs multiple transcriptional networks in medulloblastoma cells, most notably c-Myc, which is potentially involved in controlling LIFRα expression [35].

### PI3K-p110α dependent regulation of LIFRα expression

**Fig 2:**
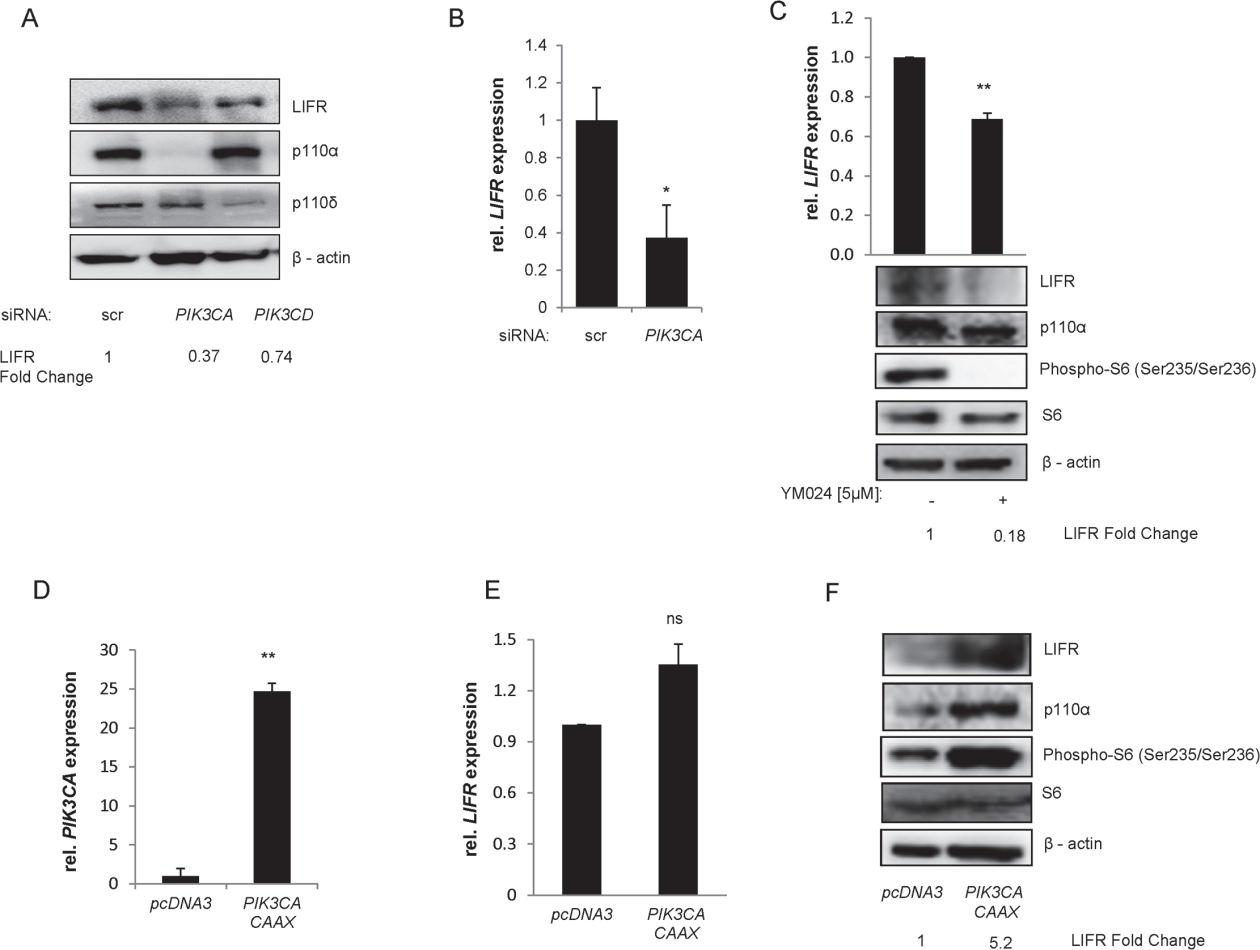
PI3K-p110α dependent regulation of LIFRα expression. Expression of LIFRα was analyzed in DAOY cells transfected with siRNA against *PIK3CA or PIK3CD* (a), at protein level by Western blot and (b) at mRNA level by RT-PCR after 48h transfection. Protein and mRNA expression of *LIFR*α was also measured upon 24h treatment with YM024 (5 μM). The expression of PI3K-p110α, total and phospho-S6 (Ser235-236) are also shown (c). pcDNA3 vector containing active PI3K-p110α (PIK3CA CAAX) or not were transfected into DAOY cells. 48h post transfection samples were analyzed for p110α and LIFRα expression by qPCR (d-e) or Western blot (f). The expression levels of total and phospho-S6 (Ser240/244) were also measured (c-f). (a, c, f) were quantified by ImageJ analysing software.

**Fig 3:**
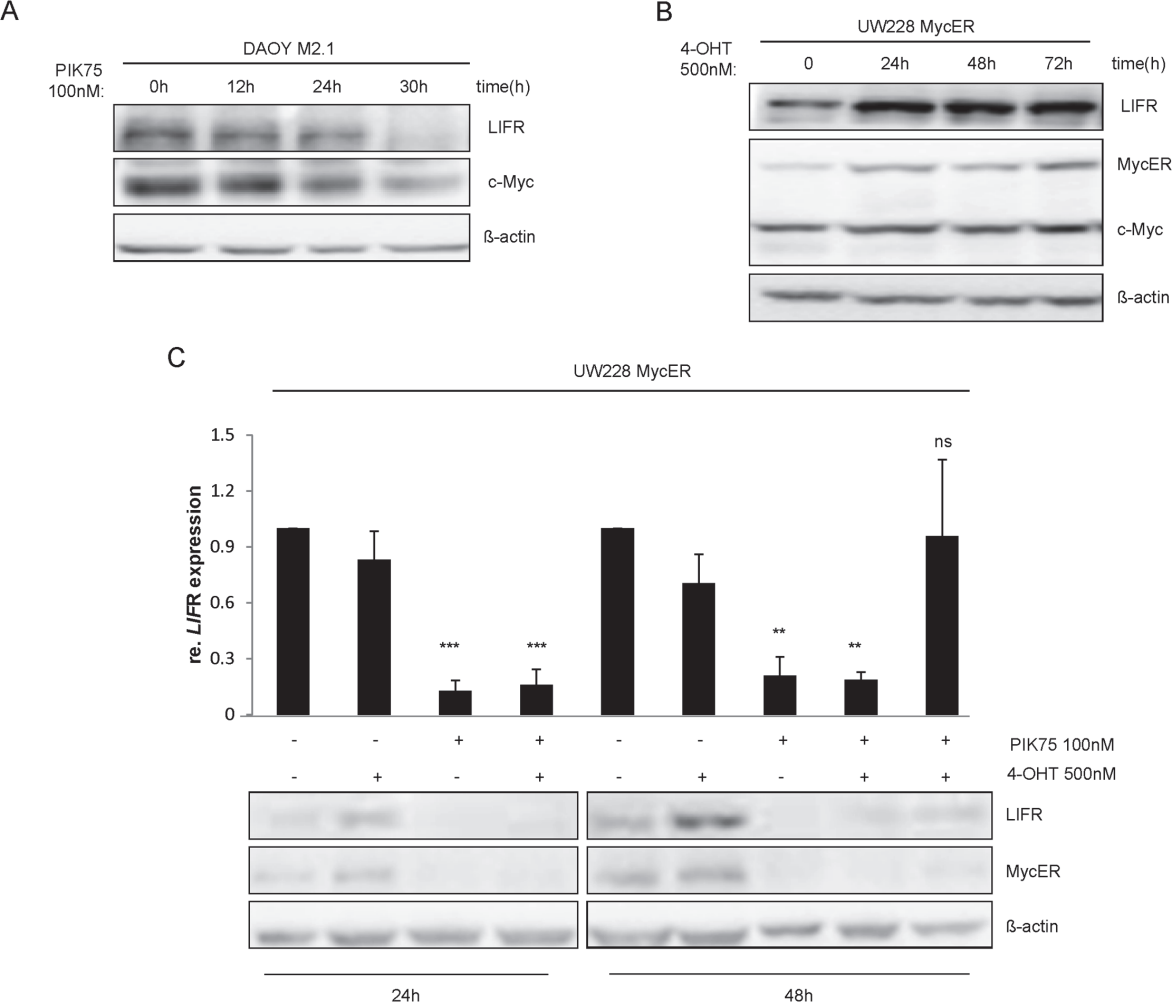
p110α regulates LIFR expression through c-Myc. The protein expression levels of c-Myc and LIFRα were evaluated after 12h, 24h and 30h of 100nM PIK75 exposure by Western blot (a) in DAOY-derived cell clones overexpressing c-Myc (DAOY M2.1) and (b) in UW228-Myc-ER cells induced for c-Myc expression by tamoxifen for 24 h, 48 h and 72 h. (c) LIFRα mRNA levels were analyzed by qPCR and protein levels were analyzed by Western blot in the inducible UW228-Myc-ER cells treated independently, simultaneously or in alternate manner with the p110α inhibitor PIK75 (100 nM) and/or with tamoxifen (500 nM) for 24 h or 48 h. UW228-Myc-ER were simultaneously exposed to both drugs for 24h (bar 4/lane 4), alternately for 48 h, bar 4/lane 4 24 h tamoxifen (500 nM) exposure followed by 24 h PIK75 (100 nM) and bar 5/lane5 24 h PIK75 (100 nM) treatment followed by 24h tamoxifen (500 nM) induction.

In order to validate the LIFRα as a bona fide target of p110α in medulloblastoma cell lines, we first used quantitative real time PCR and Western blot analysis to confirm the results obtained from the DNA microarrays. Down-regulation of the expression of the LIFRα could indeed be demonstrated at mRNA and protein level upon selective silencing of p110α, but not p110δ (Fig 2A and 2B and S3 Table). We next sought to investigate whether pharmacological inhibition of p110α also reduced LIFRα expression levels in medulloblastoma cell lines. YM024 and PIK75, two selective p110α inhibitors effectively reduced the expression levels of the receptor (Fig 2C and Fig 3A). The levels of LIFRα down-regulation achieved by treatment of DAOY with a low dose (5 μM) of YM024 were comparable to the response observed upon p110α silencing by siRNA (Fig 1 and Fig 2A–2C) and upon PIK75 (100 nM) exposure (Fig 3A and 3C). To further confirm these observations, DAOY cells were transiently transfected with active *PIK3CA* coupled with carboxy-terminal farnesylation signal allowing its localization to the membrane. Increase of p110α induced a post transcriptional up-regulation of LIFRα expression leading to activation of LIFRα downstream target, S6 (Fig 2D,2E and 2F). Collectively, these results confirm that p110α controls the expression of the LIFRα in medulloblastoma cell lines.

### Regulation of LIFR by c-Myc downstream of p110α

We next aimed to analyze the mechanism by which p110α regulates LIFRα expression. According to the analysis of transcriptional networks performed with GeneGo, c-Myc controls a network of genes comprising LIFRα (Fig 1C). To validate this network, we first analyzed the expression of LIFRα and c-Myc upon inhibition of p110α with PIK75 in a DAOY-derived cell clone, which over-expresses c-Myc. A strong inhibition of the expression of both proteins was observed upon PI3K inhibitor treatment (Fig 3A). In addition, we used the MB cell line UW228-MycER which expresses a fusion protein comprising c-Myc and the hormone-binding domain of the estrogen receptor (ER) and can be induced by tamoxifen. We observed that LIFRα expression was induced in a time-dependent manner, which paralleled with the induction of c-Myc expression (Fig 3B), previously described as a consequence of general increase in protein synthesis upon tamoxifen treatment [27, 36].

To further investigate p110α —c-Myc-dependent regulation of LIFRα, we performed simultaneous or alternate inhibition of p110α (with PIK75) and induction of c-Myc (with tamoxifen). An impaired c-Myc-dependent rescue of LIFRα protein expression was observed (Fig 3C). Interestingly, at transcriptional level, a partial rescue of the cytokine receptor was observed after 24h of PIK-75 exposure followed by 24h of c-Myc induction (Fig 3C). Together these results confirm the functionality of the gene network identified by bioinformatic analysis and describe a role for c-Myc as a regulator of LIFRα expression in MB cell lines.

### c-Myc dependent inhibition of miR-125b controls LIFRα expression

**Fig 4:**
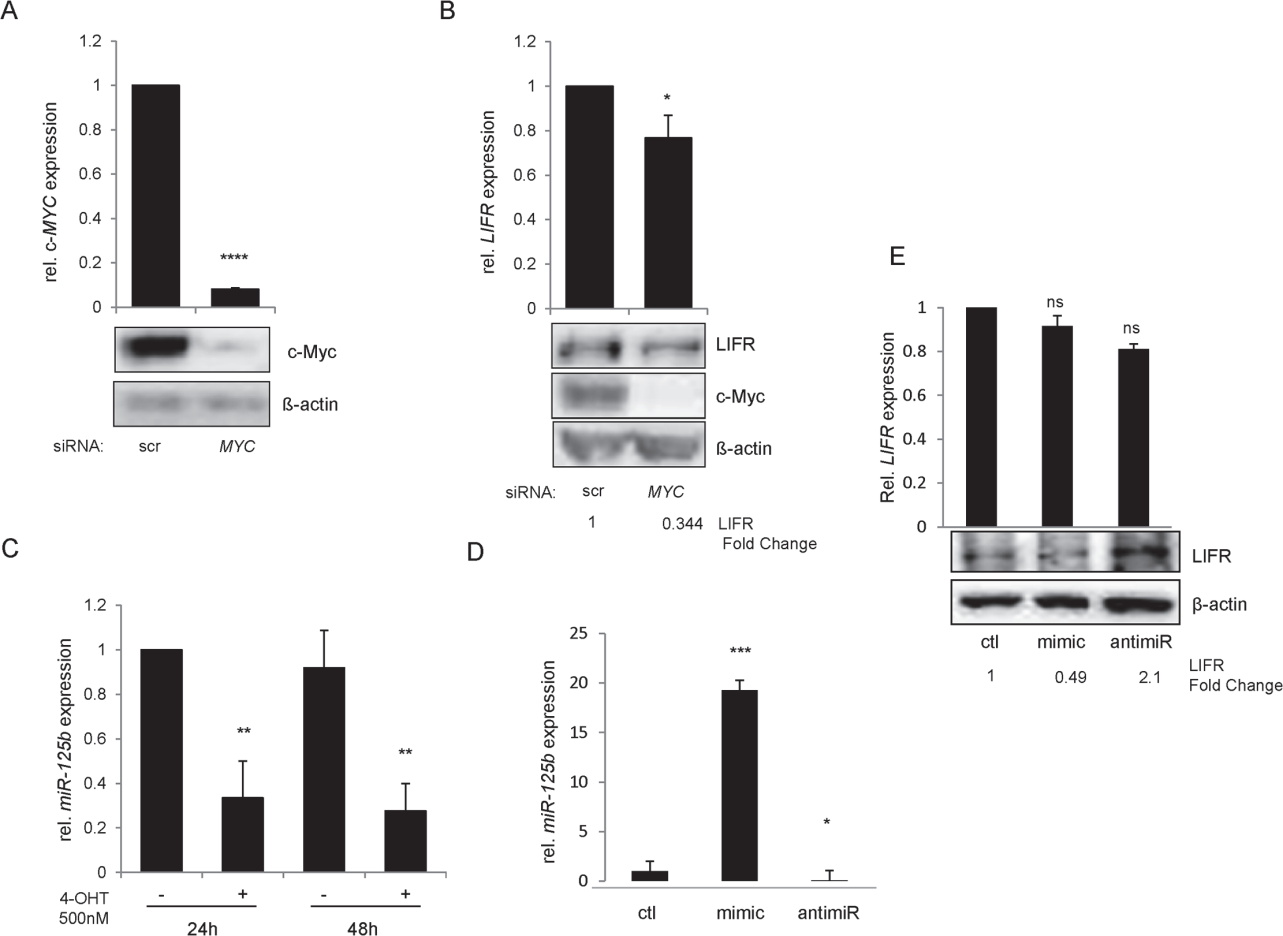
c-Myc regulates LIFR expression via miR-125b. Expression of c-Myc (a) and LIFRα (b) were analyzed by qPCR and Western Blot in DAOY M2.1 cells, overexpressing c-Myc, transfected with siRNA against c-Myc. (c) miR-125b expression levels were measured by qPCR after 24h and 48h of 4-OHT treatment in UW228-Myc-ER cells. MiR-125b fold change was calculated via normalization to U6 snRNA expression levels in the corresponding condition (d) miR-125b expression was analyzed by qPCR after transfection with mimic or antagomir for miR-125b in DAOY cells. MiR-125b fold change was calculated as in (c) (e) LIFRα expression level was analyzed by qPCR and Western blot after transfection with mimic or antagomir for miR-125b. (b) and (e) were quantified by ImageJ analysis software.

To elucidate the mechanism of the c-Myc-dependent regulation of the LIFRα, we first performed mRNA and protein expression analysis of LIFRα upon c-Myc silencing using the DAOY MB cell line over-expressing the oncogene. Silencing c-Myc induced a decrease in LIFRα protein and mRNA expression level (Fig 4B). Next, we hypothesized whether c-Myc regulates transcriptionally the expression of LIFRα by directly binding to its promoter. Possible binding sites of c-Myc on the *LIFR* promoter sequence were investigated using the software Genomatix. More than 40 possibilities were found (data not shown), providing a hint that c-Myc may bind to the promoter of *LIFR* also in medulloblastoma. However, using ChIP-on-chip analysis [24], we were unable to confirm a direct binding of c-Myc to the promoter of the *LIFR* (data not shown). Since c-Myc is well known miRNA regulator in medulloblastoma [37] and to this date LIFRα transcriptional regulation is very poorly studied we hypothesized, a c-Myc-dependent indirect control of *LIFR* expression via miRNA. Recent studies have described an auto-regulatory loop connecting the microRNA precursor of the *let-7* family with c-Myc. The oncogene transcriptionally regulates the expression of the *let-7* family members, which in turn regulate c-Myc post-transcriptionally [38]. Using the UW228 medulloblastoma cell line, expressing inducible c-Myc, we could show a significant decrease in miR-125b (a member of the *let-7* family) expression upon c-Myc induction (Fig 4C). Interestingly, another group has recently revealed that miR-125a, a miR-125b homologue, directly binds to the LIFRα 3`-UTR and inhibits its protein and mRNA expression [39, 40]. To assess the impact of miR-125b on LIFRα expression, DAOY cells were transiently transfected with the corresponding pre- or anti-miR. mRNA and protein analysis revealed miR-125b-dependent post transcriptional regulation of the receptor (Fig 4E). These results confirm the c-Myc-induced positive loop regulating LIFRα expression via inhibition of miR-125b.

### Expression of LIFRα and LIF in medulloblastoma

**Fig 5:**
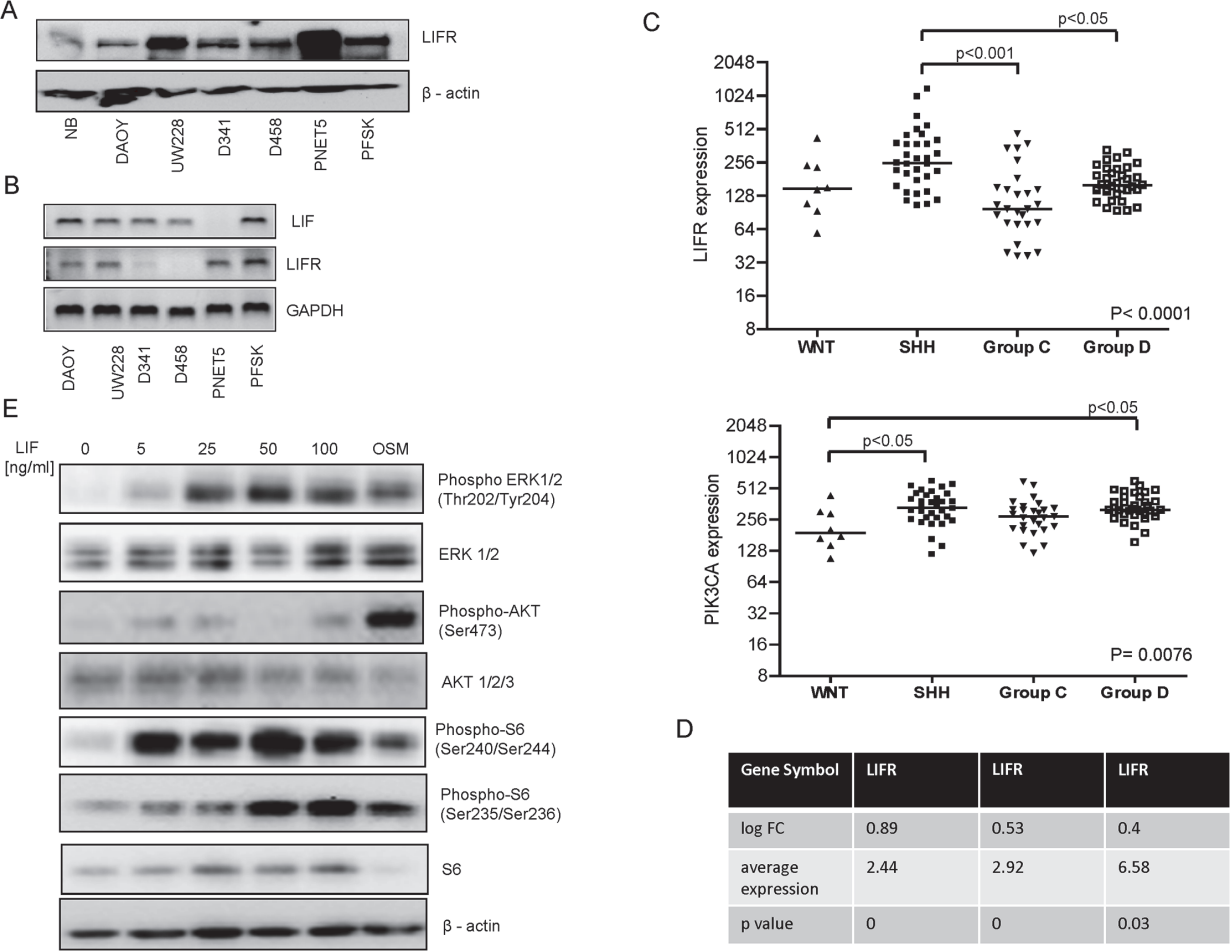
Expression of LIFRα and LIF in medulloblastoma cell lines and primary brain tumors. (a) LIFRα protein levels in medulloblastoma cell lines and normal brain (NB) tissue were evaluated by Western blot and compared with β-actin as the loading control. (b) *LIF* and *LIFR* expression in medulloblastoma cell lines was analyzed by semi quantitative RT-PCR. (c) Expression of *PIK3CA* and *LIFR* derived from the transcriptomic analysis of primary medulloblastoma, grouped according to molecular disease variants. Data from Northcott et al [32] are shown. Gene expression levels were compared using the Kruskal-Wallis test and the p-value is shown at the bottom right of the illustration. As this global test was significant, Dunn's post hoc tests were used to compare the groups. P-values < 0,05, as determined by Dunn's post hoc tests, are indicated in order to illustrate the differences. (d) Analysis of cDNA microarray data performed on *PTCH1* ^*+/-*^; *TP53* ^*+/-*^ or *PTCH1* ^*+/-*^ *; TP53* ^*+/+*^ mice. The table represents a summary of fold change (logFC), average expression values (AveExpr), p values obtained by comparison of the two murine strains from Data set GEO accession number GSE37316). (e) Cell lysates of DAOY cells stimulated with or not with 5, 10, 25, 50 and 100 ng/ml of LIF for 10 min were analyzed for expression and phosphorylation of AKT and ERK pathway downstream targets. Treatment of 10 ng/ml of OSM for 10 min was used as a positive control.

Our previous work had documented an over-expression of p110α in primary medulloblastoma, as well as in medulloblastoma cell lines [15]. Therefore, we hypothesized that the LIFRα may also be over-expressed in primary medulloblastoma. A panel of medulloblastoma lines was screened for LIFRα expression by Western blot analysis, and increased expression of the cytokine receptor was observed in all cell lines when compared to normal brain (Fig 5A). PNET5 and UW228 presented the most increased LIFRα expression level among the MB cell lines. The expression analysis at mRNA level showed that 4 out of 7 cell lines expressed detectable levels of LIFRα mRNA (Fig 5B).

We next sought to investigate whether LIFRα was co-expressed with its ligand LIF in MB cell lines. Three cell lines (DAOY, UW228 and PFSK) co-expressed both the ligand and the receptor (Fig 5A and 5B), indicating the presence of an autocrine loop in a subset of medulloblastoma cell lines.

In order to investigate the clinical relevance of LIFRα, its expression was also analyzed in the cancer microarray database Oncomine [41]. Data obtained from a published study by Pomeroy et al. [42] which compared gene expression profiles of different cohorts of classic and desmoplastic medulloblastomas, supratentorial primitive neuroectodermal tumor, atypical teratoid/rhabdoid tumor and glioblastoma vs. normal cerebellum, showed that the *LIFR* was over-expressed in classic medulloblastoma, demosplastic medulloblastoma and in glioblastoma when compared to normal cerebellum. (S1 Fig).

Next, we investigated the expression levels of *PIK3CA* and *LIFR* in two primary medulloblastoma cohorts published by Kool et al. [31] and Northcott et al. [32]. *LIFR* presented lower expression levels in group WNT and Group 3, while in SHH and Group 4 *LIFR* displayed higher expression levels. This unique expression was confirmed in both medulloblastoma cohorts [31, 32]. The non-WNT tumors (group SHH, group 3 and 4) were characterized by highest *PIK3CA* expression in comparison to patients with good prognosis (group WNT) presenting lower *PIK3CA* expression (Fig 4C). *PIK3CA* and *LIFR* expressions correlated exclusively in the WNT and SHH subgroups, lower and higher expression correspondingly (Fig 5C and S2 Fig).

To further investigate the impact of *LIFR* expression in medulloblastoma tumors, we compared two cDNA microarray data performed on heterozygous for Patched receptor mice, expressing wild type or single copy of *TP53* [43, 44]. Interestingly, in the SHH MB mimicking murine model (*PTCH*-/+, *TP53* -/+), *LIFR* expression was significantly higher, confirming the role of the cytokine receptor in these tumors (Fig 5D and S3 Fig).

### LIFRα activation stimulates multiple intracellular signaling pathways in medulloblastoma

In order to investigate whether the LIFRα is functional in medulloblastoma cell lines, we analyzed the activation status of downstream signaling pathways in response to cell stimulation with LIF. Dose-dependent activation of ERK and AKT pathways, leading to activation via phosphorylation of their downstream target S6 was detected, starting at a concentration of LIF of 5ng/ml and with a maximal effect at 100ng/ml in DAOY cells (Fig 5E). Furthermore, activation of downstream signaling pathways in response to cell stimulation with oncostatin M (OSM), a potent activator of LIFR signaling confirmed our data (Fig 5E and S4 Fig). In view of the activation of these classical downstream signaling pathways, it can be concluded that the LIFRα is indeed functional in medulloblastoma cell lines.

### Targeting LIFRα signaling in medulloblastoma

**Fig 6:**
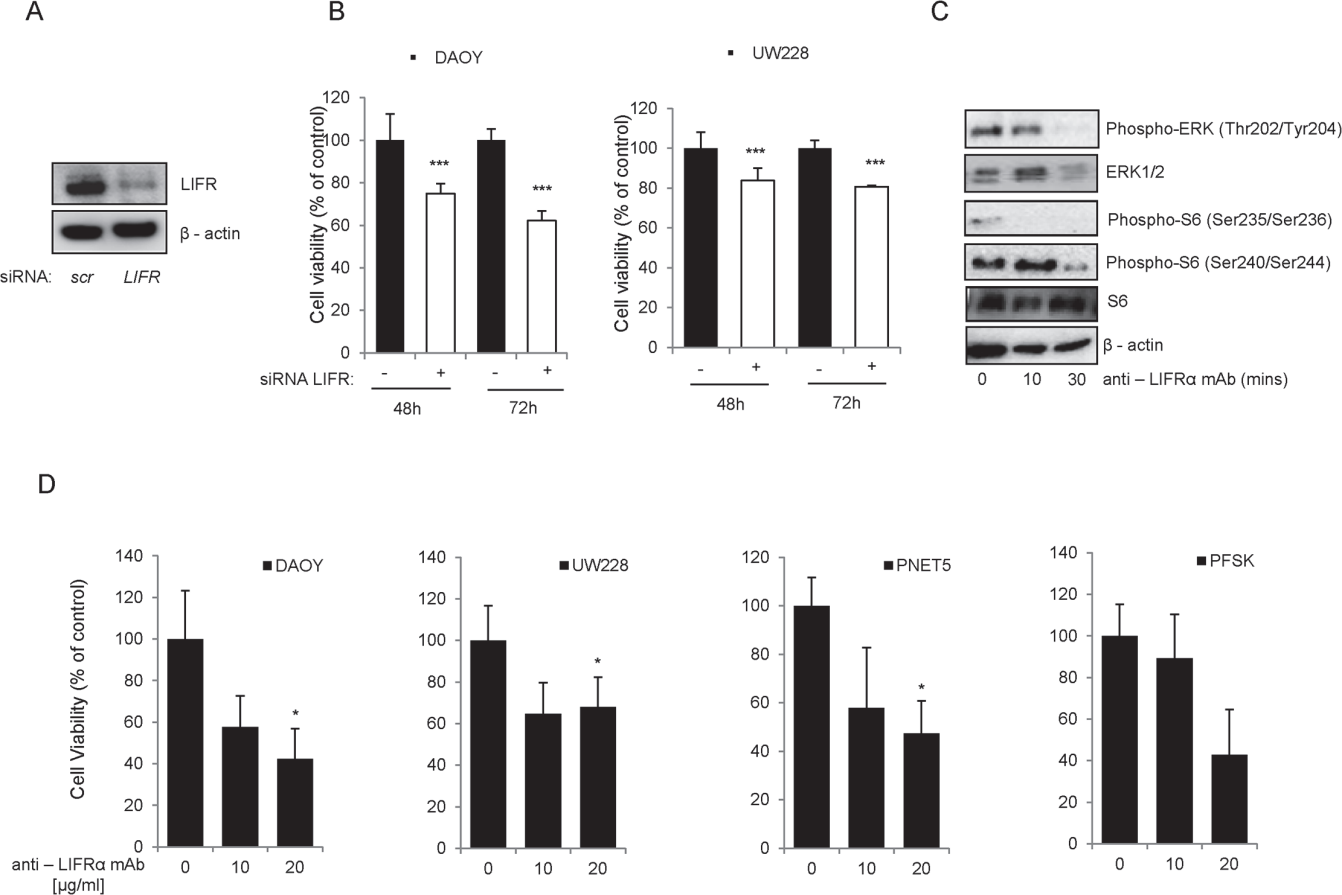
Effects of LIFRα down-regulation on medulloblastoma cell proliferation and survival. (a) Protein expression level of LIFRα in DAOY cells transfected with siRNA against *LIFR* compared to the control siRNA transfected cells. (b) The effects on cell viability of the siRNA-mediated target down-regulation of LIFRα (white bars) compared to control siRNA (black bars) in DAOY and UW228 cells was assessed for the indicated time points and expressed as percentages. (c) The protein expression of LIFRα downstream targets was analyzed upon treatment for 10 or 30 min with 20μg/ml of anti-LIFRα neutralizing antibody. β-actin was used as a loading control. (d) Cell viability was evaluated in DAOY, UW228, PNET5 and PFSK cells treated for 48h with an anti-LIFRα neutralizing antibody at the indicated concentrations.

The role of LIFRα in the control of medulloblastoma cell proliferation and survival was investigated by RNAi downregulation and pharmacological inhibition with a neutralizing antibody against the receptor and subsequent analysis of MB cell responses. We first used siRNA to transiently down-regulate the expression of the LIFRα in medulloblastoma cell lines (Fig 6A). Down-regulation of the cytokine receptor led to significantly decreased cell proliferation in DAOY and UW228 cell lines (Fig 6B).

**Fig 7:**
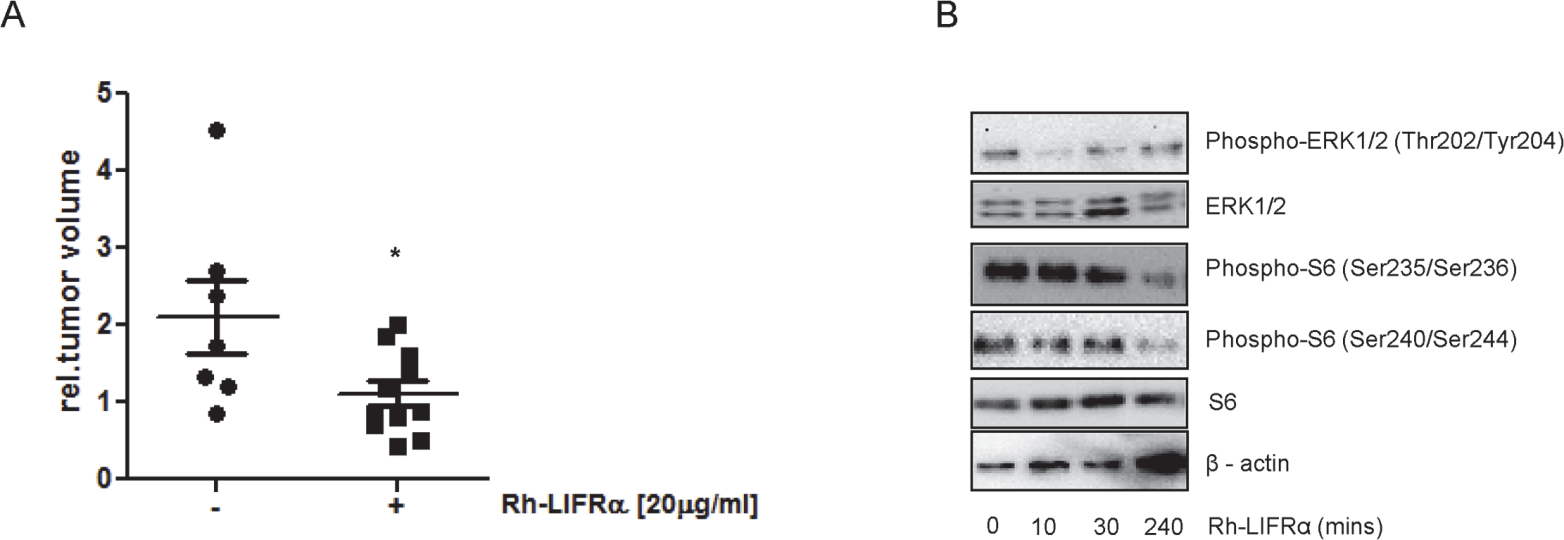
LIFRα inhibition induces reduction in Medulloblastoma tumor volume. (a) Tumors formed on (CAM) were treated with 20μg/ml of rh-LIFα recombinant or phosphate-buffered saline for 4 consecutive days. Quantification of tumor volume changes before and after treatment is shown. Lines indicate the mean of each group, *P<0.05 compared with control treatment. (b) The expression of LIFRα downstream targets was analyzed upon treatment for 10, 30, 240 min with 20 μg/ml of rh-LIFRα. β-actin was used as a loading control.

Furthermore, we analyzed the effect of the cytokine receptor inhibition on the downstream targets of the signaling pathway. Time-dependent inhibition of ERK and AKT pathways, leading to decreased phosphorylation of their downstream target the ribosomal protein S6, was measured upon treatment of 20μg/ml of LIFRα neutralizing antibody in DAOY cells (Fig 6C). This indicates that the neutralizing antibody disrupts LIF/LIFRα signaling. Similar results were obtained when using a recombinant human soluble LIFRα as a neutralizing reagent (Fig 7B). These results were confirmed in 4 MB cell lines, where upon exposure to LIFRα neutralizing antibody, a significant decrease in cell proliferation was observed (Fig 6D).

In order to explore the therapeutic potential of LIFRα inhibition *in vivo*, we performed a chicken embryo chorioallantoic membrane assay (CAM). Direct application of DAOY cells onto the chorioallantoic membrane led to the formation of solid tumors within 3 days. For the following 4 days, the tumors were treated with 20μg/mL of recombinant human soluble LIFRα as a neutralizing reagent (ligand trap) and their volume was compared to a control treatment. As expected, a significant reduction in the tumor volume was observed upon inhibition of LIF/LIFRα (Fig 7A). Confirmation of the efficacy of the recombinant human soluble LIFRα, was observed in DAOY cells treated for 10, 30 and 240 minutes with 20μg/mL of the neutralizing reagent. As expected, similarly to LIFRα neutralizing antibody, the recombinant human LIFRα inhibited the AKT and ERK pathways leading to decreased phosphorylation of the ribosomal protein S6 (Fig 7B). Together these findings confirm the key involvement of LIFRα in medulloblastoma tumorigenesis.

## Discussion

The PI3K/Akt pathway has been demonstrated to play a key role in medulloblastoma cell proliferation, survival, chemoresistance and migration [4, 11, 15, 16, 45, 46]. Mutations in *PIK3CA* were reported in primary medulloblastoma [23], as well as increased expression of *PIK3CA* at the mRNA and protein level [15]. Isoform-specific or broad specificity PI3K inhibitors have shown anti-tumor activity in medulloblastoma models *in vitro* and *in vivo* [4, 15, 16]. In general, these agents reduced the activation status of classical PI3K downstream targets, such as Akt, mTOR, S6K or GSK-3β.

Targeting p110α by RNAi or isoform-specific inhibitors had more pronounced effects on medulloblastoma cell responses than in the case of p110β or p110δ, indicating a selective role for p110α in medulloblastoma [15]. This hypothesis was also confirmed by the observation that *PIK3CA* was over-expressed in primary medulloblastoma, compared to normal cerebellum, which was not the case for *PIK3CB* or *PIK3CD* [15]. We have previously shown that while p110δ knock-down by siRNA does not impair cell proliferation in MB, co-targeting p110α and p110δ led to a greater reduction cell proliferation than single p110alpha targeting [15]. The precise role of p110δ in MB however remains to be clarified.

In view of these observations, we hypothesized that p110α may control the expression of a selective subset of genes implicated in medulloblastoma cell proliferation and/or survival. The comparative DNA microarray analysis of medulloblastoma cell lines in which either p110α or p110δ were down-regulated by siRNA identified such a gene subset.

The LIFRα was validated as a downstream target of p110α by a combination of approaches, including pharmacological inhibition or over-expression of the class I_A_ PI3K isoform. The observation that *LIFR*α expression was elevated in the SHH subgroup of primary medulloblastoma, further supports this model, in view of the over-expression of *PIK3CA* in this subtype.

Importantly, the LIFRα was shown to be functional in medulloblastoma cell lines, since its activation stimulated classical downstream signaling pathways, such as JAK/STAT, Erk1/2 and PI3K/Akt. Moreover, the observation that medulloblastoma cell lines generally express LIF confirms the presence of an autocrine loop in this malignant brain tumor [47]. LIF is a pleiotropic cytokine, which can sustain proliferation or differentiation depending on cell type or maturation [48]. Deregulated LIF secretion has also been previously described in human cancer, such as breast cancer, rhabdomyosarcoma and medulloblastoma [47, 49, 50]. In medulloblastoma, cell lines, LIF expression was reported to be regulated by p53 [51]. Interestingly, we have shown a p53-dependent negative regulation of the receptor expression in *PTCH*-/+ mice expressing wild type *TP53* compared to mice expressing heterozygous *TP53*. Considering the previously published LIF dependent negative feedback loop on *LIFRα* expression [52], the ensemble of these data strongly suggest an indirect p53-dependent regulation of *LIFR*α via LIF.

To our knowledge, we performed for the first time *in vivo* inhibition of LIFRα which let to tumor volume reduction in CAM assay model. Targeting the LIFRα downstream signaling has also been evaluated in medulloblastoma. Interfering with STAT3 by using a non-peptide small molecule STAT3 inhibitor was reported to decrease cell viability and induce apoptosis in medulloblastoma [39]. A study using resveratrol also reported on the importance of STAT3 signaling in maintenance and survival of medulloblastoma cells [53].

There exist so far few reports on the regulation of LIFRα expression and promoter activity. In breast cancer, c-Myc was shown to act as a negative regulator of *LIFR*α expression [35]. In MB, we failed to detect a direct binding of c-Myc to the promoter of the receptor. Nevertheless, c-Myc-dependent, multiple-partners transcriptional regulation of LIFRα remains plausible. However, our data support a model in which c-Myc indirectly controls LIFRα protein expression by repressing miR-125b, a negative regulator of the cytokine receptor. In this regard, recent studies have revealed down-regulation of miR-125b in primary medulloblastoma tumors compared to normal fetal brain [54]. Furthermore, the LIFRα-dependent proliferation increase in our MB cell lines, tumor volume reduction upon LIFRα inhibition *in vivo* and the high LIFRα expression levels in the SHH subgroup of MB, are supported by data published by Ferretti at al. [55] documenting miR-125b-dependent inhibition of cerebellar progenitor proliferation, via negative regulation of Smoothened, an upstream component in the SHH pathway. Interestingly, data from Peng et al have described the microRNA as prognosis biomarker in oral cavity squamous cell carcinoma [56] suggesting strongly the measurement of the circulating miR-125b also in medulloblastoma. Furthermore, our results suggest PI3K-dependent transcriptional regulation of LIFR independently of Myc. Up to date, there are no data describing direct interaction between the cytokine receptor promoter and a transcription factor.

Previous studies have reported on cross-talks between the PI3K/Akt pathway and *myc*-family genes. GSK-3β (glycogen synthase kinase 3, isoform β), which is directly regulated by phosphorylation through Akt controls Myc phosphorylation and stability [12, 57]. An additional mechanism of PI3K-dependent regulation of Myc is through PDK1 (Phosphoinositide-dependent kinase-1). The latter phosphorylates PLK1 (Polo-like kinase 1) which in turn induces MYC phosphorylation at Ser62 and promotes cell growth and survival [58]. In Medulloblastoma, treatment with the PDK1 inhibitor OSU03012 induced apoptosis *in vitro* and inhibited xenograft tumor growth [4].

In embryonal tumors, including medulloblastoma, targeting the AKT and ERK pathways using a quassinoid analogue induced c-Myc and N-myc down-regulation [7, 59]. Inhibition of PI3K using a selective inhibitor was also reported to induce N-myc down-regulation in neuroblastoma [60]. N-myc was shown to play an important role in the regulation of PI3K-mediated VEGF secretion in neuroblastoma cells [61, 62]. Interestingly, *MYCN* is often found amplified in SHH type of Medulloblastoma and its expression is linked to poor patient outcome [5, 63]. Recently, *MYC* amplification has been reported to be implicated in the resistance of tumors with activated *PIK3CA* to pharmacological inhibitors of p110α [40, 64].

Together our results delineate a novel signaling pathway from p110α to c-Myc, mir-125b and LIFRα, which contributes to key medulloblastoma cell responses and may be further studied to develop novel targeted therapies for this common and devastating childhood malignancy.

## Supporting Information

S1 Fig LIFR expression in primary brain tumors. *LIFR* gene expression profile in different cohorts of atypical teratoid/rhabdoid tumour (1), classic (2) and desmoplastic (3) medulloblastomas, glioblastoma (4) and primitive neuroectodermal tumor (5) compared to normal cerebellum (0). Due to large variations in sample sizes, statistical analysis was not performed. The fold changes (FC) in expression are listed as a reference for this analysis (FC _Malignant Glioma_ = 1.53_,_ FC _Desmoplastic Medulloblastoma_ = 1.3_,_ FC _Classic Medulloblastoma_ = 1.19_,_ FC _AT/RT_ = -1.8). Data were analyzed with www.oncomine.org, based on Pomeroy et al (27).
(TIF)

S2 Fig Expression of LIFR and PIK3CA in primary medulloblastoma subtypes. Expression of *PIK3CA* and *LIFR* derived from the transcriptomic analysis of primary medulloblastoma, grouped according to molecular disease variants. Data from Kool et al. (28) are shown.
(TIFF)

S3 Fig LIFRα expression in mouse-driven model of SHH medulloblastoma. Microarray analysis of LIFRα expression in murine PTCH+/- p53+/- cancer stem cells compared to PTCH+/- p53+/+ cells (Data set GEO accession number GSE37316). Volcano plot of fold changes (log2 scale) versus p values (-1log10 scale, grey) and adjusted p values (-log10 scale, pink) is shown. Highlighted in red are the values for various probe sets representing LIFR.
(TIF)

S4 Fig LIFRα signaling in medulloblastoma. Cell lysates of DAOY cells stimulated with oncostatin M (OSM) for 10 min were analyzed for expression and phosphorylation of the indicated OSM downstream targets.
(TIFF)

S1 Table List of PI3K-p110α regulated genes in DAOY cells. List of a gene set deregulated upon *PIK3CA* silencing in DAOY cells.
(TIFF)

S2 Table List of PI3K-p110δ regulated genes in DAOY cells. List of a gene set deregulated upon *PIK3CD* silencing in DAOY cells.
(TIFF)

S3 Table Analysis of transcriptional networks by GeneGo Metacore. Hit list of the transcription factors whose gene networks were mostly affected by the RNAi mediated downregulation of *PIK3CA* and *PIK3CD* in DAOY cells.
(TIF)

S4 Table Genes involved in c-Myc transcriptional network. Fold changes in the gene expression of predicted c-Myc target genes in DAOY cells transfected with the indicated siRNAs (targeting *PIK3CA* or *PIK3CD*) versus control siRNA are listed.
(TIFF)
