# Supplementary figures and images for: The Phosphoinositide 3-Kinase p110α Isoform Regulates Leukemia Inhibitory Factor Receptor Expression via c-Myc and miR-125b to Promote Cell Proliferation in Medulloblastoma
Source: PLoS One. 2015 Apr 27;10(4):e0123958. doi: 10.1371/journal.pone.0123958 (PMC4411098; doi:10.1371/journal.pone.0123958)

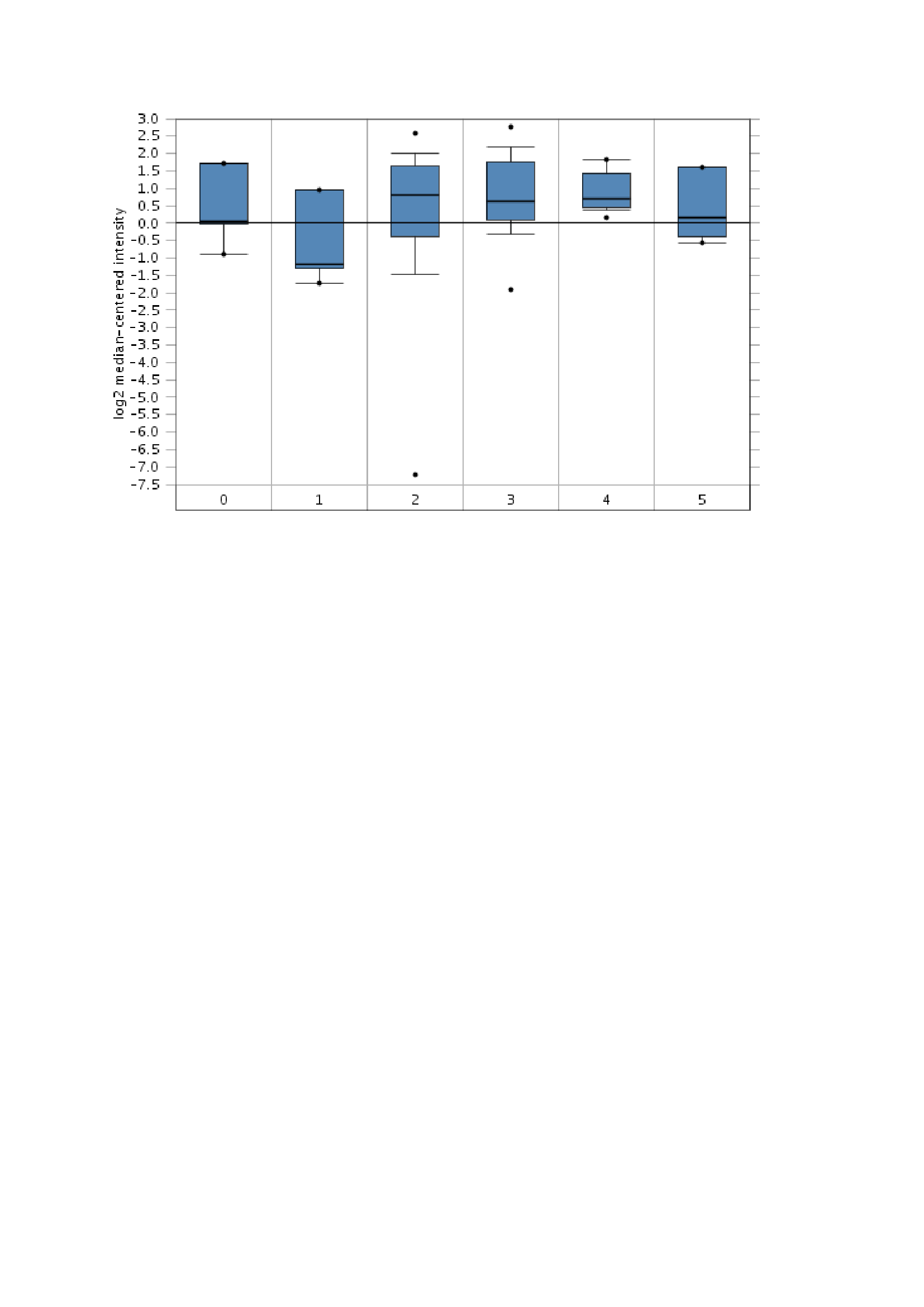

Supplement: S1 Fig — LIFR gene expression profile in different cohorts of atypical teratoid/rhabdoid tumour (1), classic (2) and desmoplastic (3) medulloblastomas, glioblastoma (4) and primitive neuroectodermal tumor (5) compared to normal cerebellum (0). Due to large variations in sample sizes, statistical analysis was not performed. The fold changes (FC) in expression are listed as a reference for this analysis (FC Malignant Glioma = 1.53, FC Desmoplastic Medulloblastoma = 1.3, FC Classic Medulloblastoma = 1.19, FC AT/RT = -1.8). Data were analyzed with www.oncomine.org, based on Pomeroy et al (27). (TIF) [file pone.0123958.s001.tif]

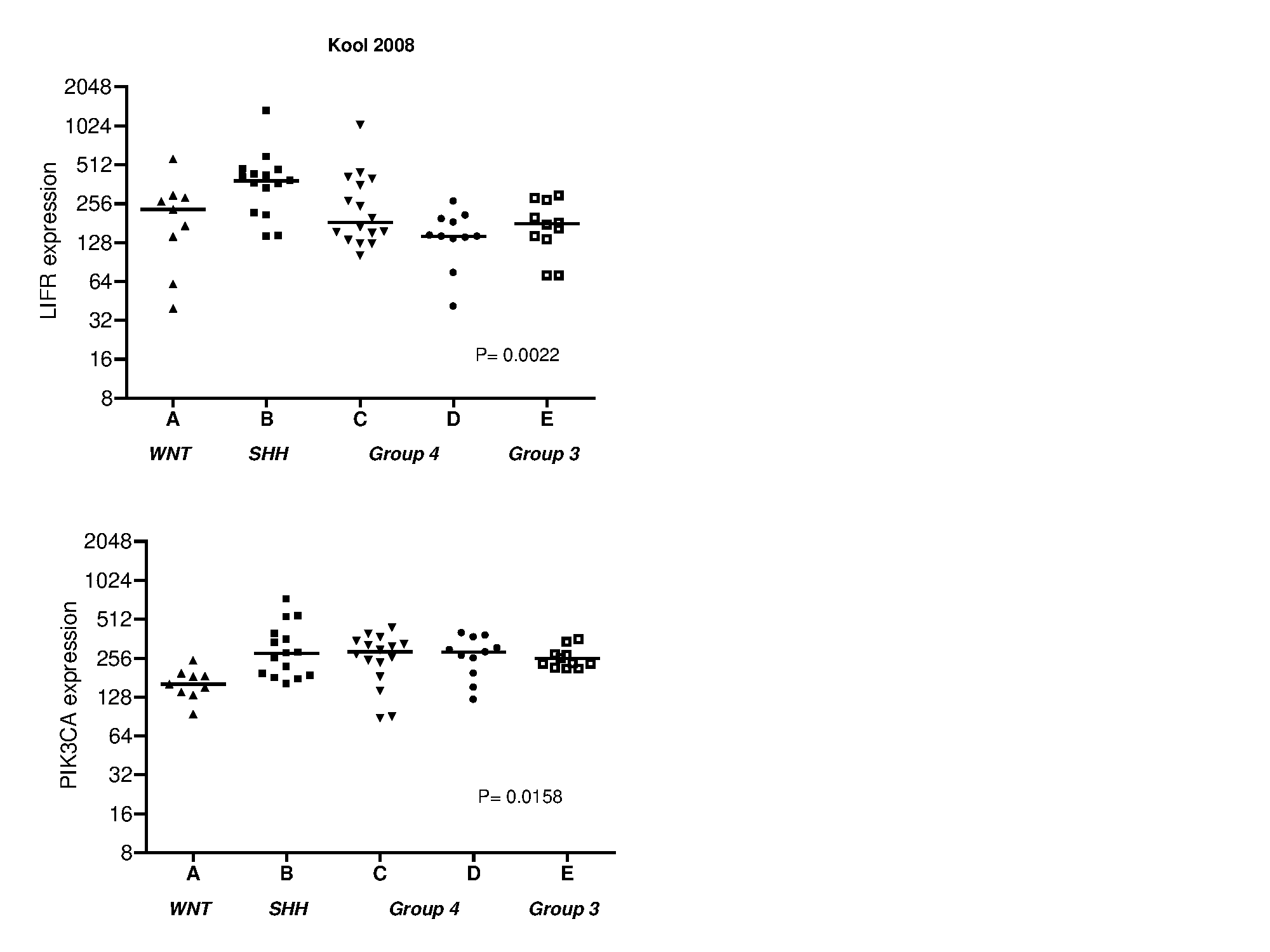

Supplement: S2 Fig — Expression of PIK3CA and LIFR derived from the transcriptomic analysis of primary medulloblastoma, grouped according to molecular disease variants. Data from Kool et al. (28) are shown. (TIFF) [file pone.0123958.s002.tiff]

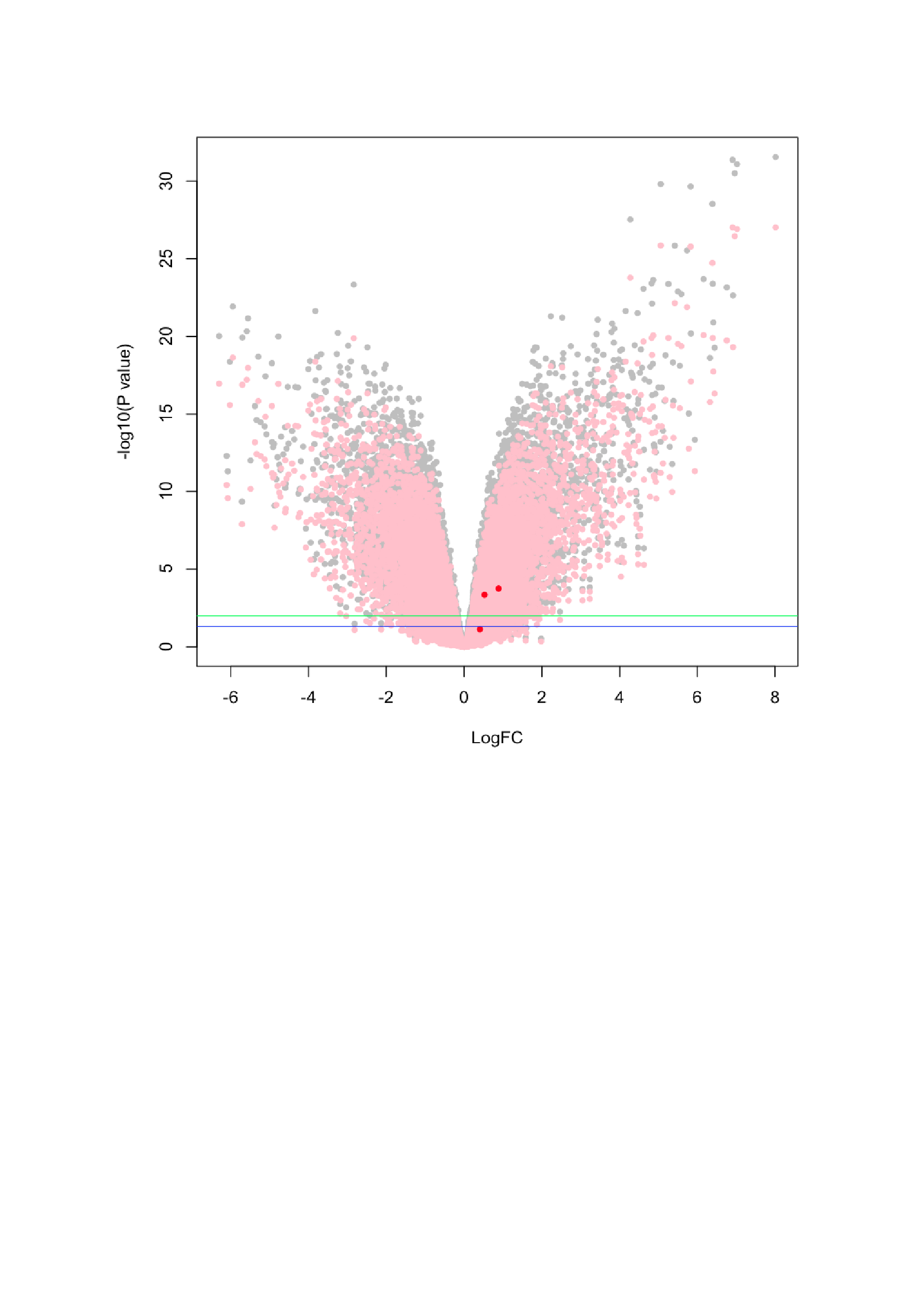

Supplement: S3 Fig — Microarray analysis of LIFRα expression in murine PTCH+/- p53+/- cancer stem cells compared to PTCH+/- p53+/+ cells (Data set GEO accession number GSE37316). Volcano plot of fold changes (log2 scale) versus p values (-1log10 scale, grey) and adjusted p values (-log10 scale, pink) is shown. Highlighted in red are the values for various probe sets representing LIFR. (TIF) [file pone.0123958.s003.tif]

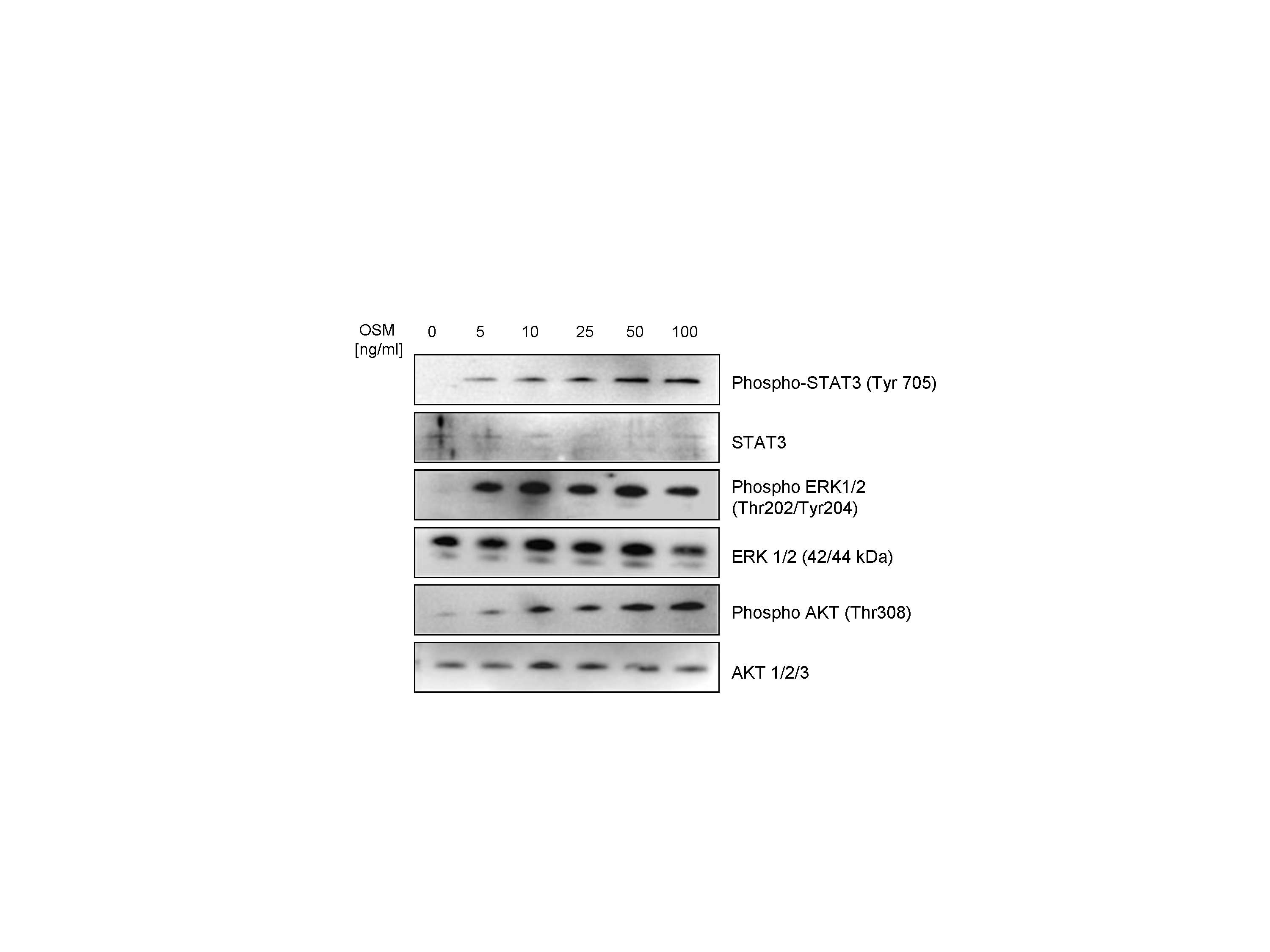

Supplement: S4 Fig — Cell lysates of DAOY cells stimulated with oncostatin M (OSM) for 10 min were analyzed for expression and phosphorylation of the indicated OSM downstream targets. (TIFF) [file pone.0123958.s004.tiff]

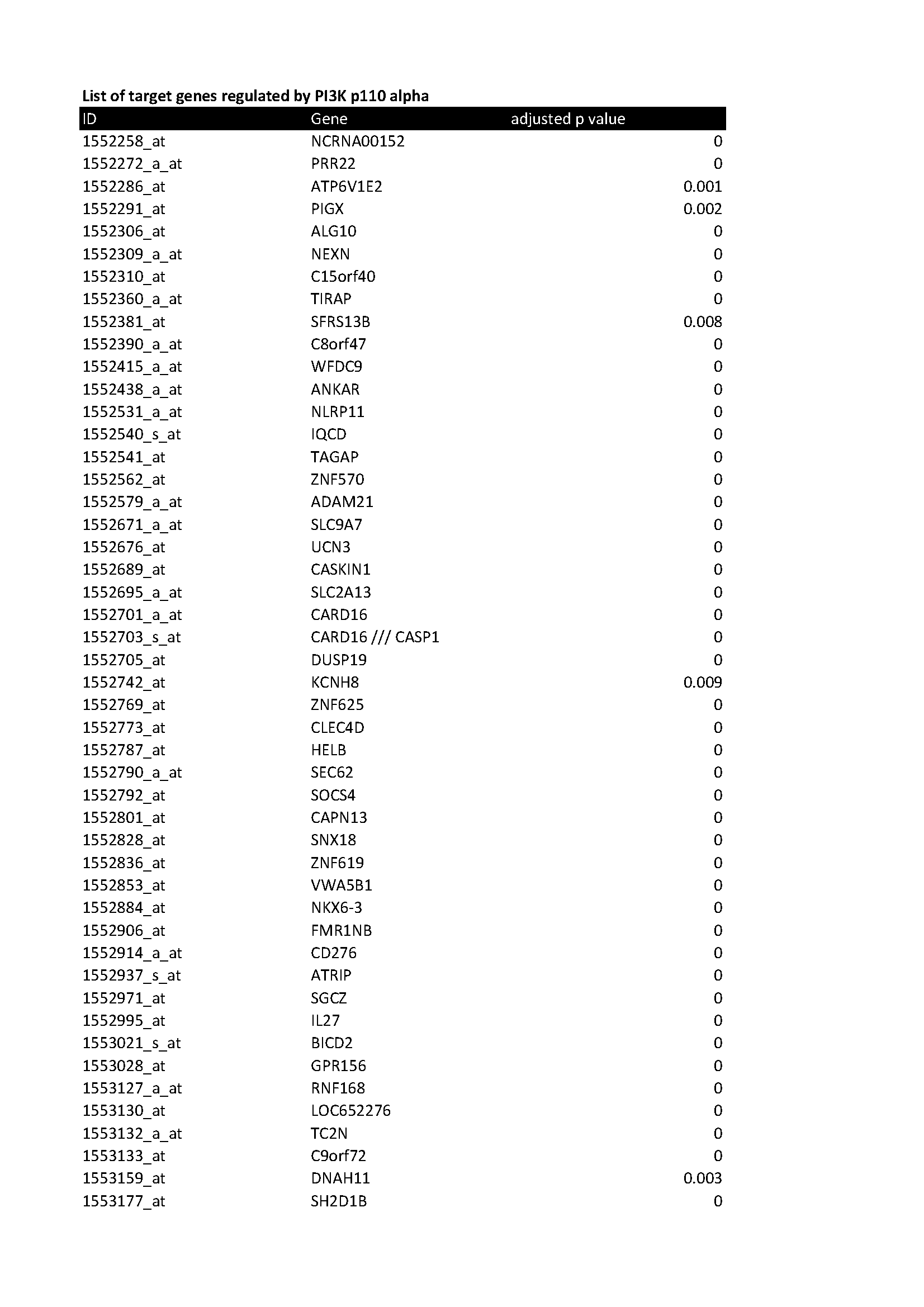

Supplement: S1 Table — List of a gene set deregulated upon PIK3CA silencing in DAOY cells. (TIFF) [file pone.0123958.s005.tiff]

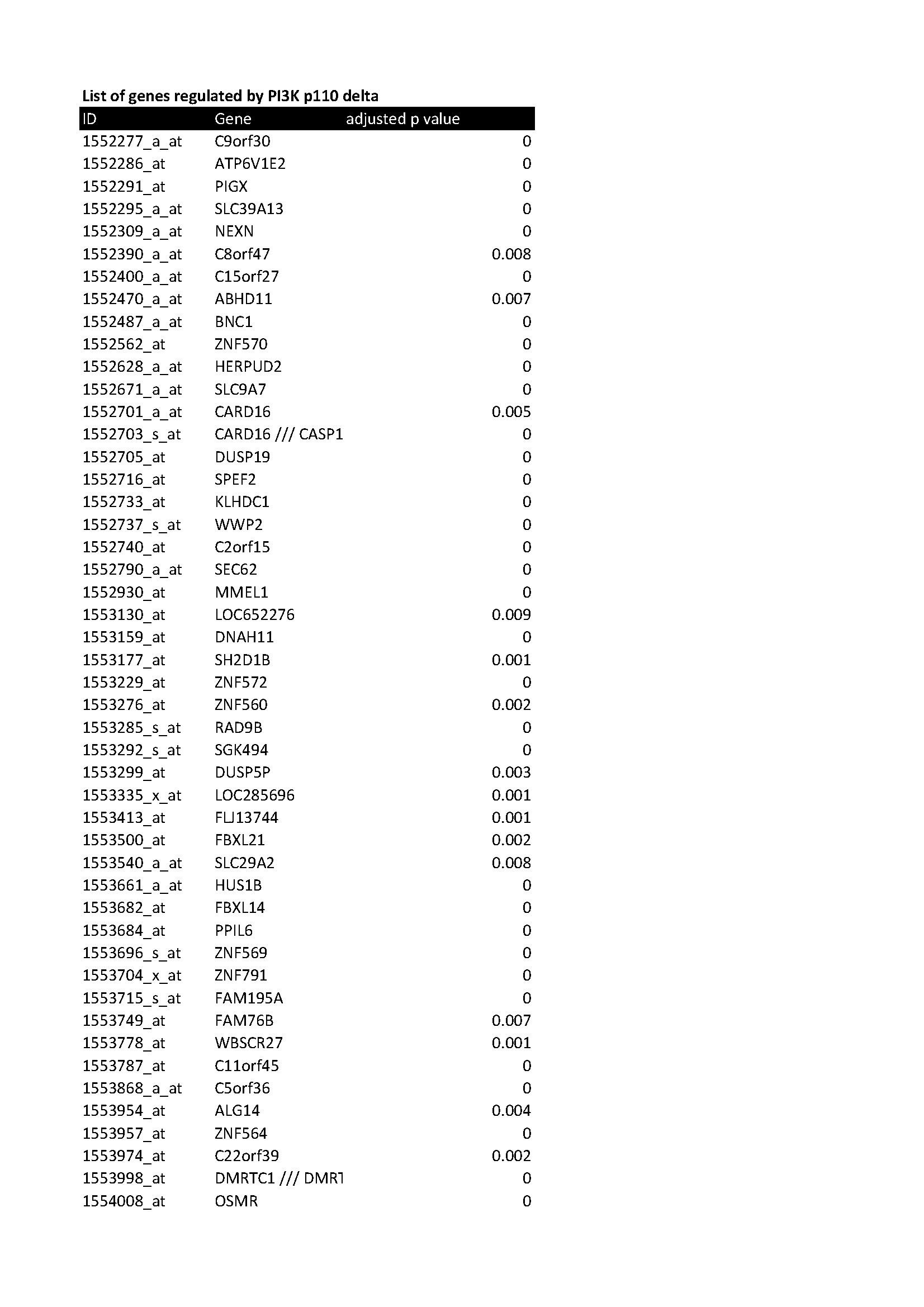

Supplement: S2 Table — List of a gene set deregulated upon PIK3CD silencing in DAOY cells. (TIFF) [file pone.0123958.s006.tiff]
